# Evaluation of Cryogen-Free Thermal Modulation-Based Enantioselective Comprehensive Two-Dimensional Gas Chromatography for Stereo-Differentiation of Monoterpenes in *Citrus* spp. Leaf Oils

**DOI:** 10.3390/molecules28031381

**Published:** 2023-02-01

**Authors:** Haneen Ibrahim Al Othman, Atiqah Zaid, Francesco Cacciola, Zhijun Zhao, Xiaosheng Guan, Jalal T. Althakafy, Yong Foo Wong

**Affiliations:** 1 Centre for Research on Multidimensional Separation Science, School of Chemical Sciences, Universiti Sains Malaysia, Penang 11800, Malaysia; 2 Department of Biomedical, Dental, Morphological and Functional Imaging Sciences, University of Messina, 98125 Messina, Italy; 3 J&X Technologies (Shanghai) Co., Shanghai 200438, China; 4 Department of Chemistry, Faculty of Applied Science, Umm Al-Qura University, Makkah 21955, Saudi Arabia

**Keywords:** chiral gas chromatography, comprehensive two-dimensional gas chromatography, monoterpenes, *Citrus hystrix*, *Citrus pyriformis*, *Citrus limon*, *Citrus microcarpa*

## Abstract

This study evaluates the applicability of enantioselective gas chromatography (*e*GC) and enantioselective comprehensive two-dimensional gas chromatography (*e*GC×GC) coupled with flame ionization detection for the stereospecific analysis of designated chiral monoterpenes within essential oils distilled from the leaves of *Citrus hystrix* (CH), *C. limon* (CL), *C. pyriformis* (CP), and *C. microcarpa* (CM). A cryogen-free solid-state modulator with a combination of enantioselective first-dimension and polar second-dimension column arrangements was used to resolve potential interferences in *Citrus* spp. leaf oils that can complicate the accurate determination of enantiomeric compositions. Interestingly, considerable variations were observed for the enantiomeric fractions (EFs) of the chiral terpenes. (+)-limonene was identified as the predominant enantiomer (60.3–98.9%) in all *Citrus* oils, (+)-linalool was the major enantiomer in CM (95.9%), (−)-terpenin-4-ol was the major isomer in CM (66.4%) and CP (61.1%), (−)-α-pinene was the dominant antipode in CL (55.5%) and CM (92.1%). CH contained (−)-citronellal (100%) as the pure enantiomer, while CL and CP have lower proportions (9.0–34.6%), and citronellal is absent in CM. The obtained enantiomeric compositions were compared and discussed with results from *e*GC using the same enantioselective column. To our knowledge, this work encapsulates the first report that details the EFs of these chiral monoterpenes in *Citrus* spp. leaf oil.

## 1. Introduction

The genus *Citrus*, belonging to the Rutaceae family, is represented by about 160 genera with 16 (Swingle System; [1]) to 162 (Tanaka System; [2]) species that are distributed throughout tropical and subtropical regions worldwide [3]. To date, *Citrus* has been much appreciated as one of the most important commercial fruit crops, with an estimated global production of lemons and limes above 9 million tons during the period of 2021–2022 [4]. In particular, the aromatic oil isolated from the fruit peels (commonly known as *Citrus* essential oil (EO)) of various citrus trees is highly valued in the pharmaceutical, food, and perfume industries [5,6]. *Citrus* spp. EOs have been reported to exhibit a wide spectrum of biological activity, including anti-carcinogenic, anti-bacterial, antioxidant, anti-fungal, anti-microbial, anti-spasmodic, anti-diabetic, anti-dermatophyte, and anti-inflammatory activities [7,8,9,10].

Chemically, *Citrus* EO constitutes a complex pool of bioactive secondary compounds that vary depending on their species, variety, cultivar, origin, climate, and others [11,12,13,14]. These aromatic *Citrus* oils normally consist predominantly of a complex mixture of monoterpenes and sesquiterpenes, whose relative concentrations vary according to species. Notably, the demand for genuine *Citrus* spp. EOs is continuously increasing worldwide, with selected *Citrus* spp. oils fetching higher market values (e.g., *Citrus hystrix* EO (>150 USD per kg) compared to *Citrus sinensis* EO (3.5–5.0 USD per kg) [15,16]. Consequently, many reports have been made on the occurrences of mislabeling or adulteration incidents, such as blending with other inexpensive EOs or the addition of lower-cost synthetic components [17,18,19,20]. In light of these phenomena, many analytical approaches (e.g., gas chromatography, isotope ratio mass spectrometry, and others) have been developed to authenticate and safeguard the quality of *Citrus* EOs [21,22,23,24].

It is known that plant terpenes are biosynthesized via a series of biogenetic pathways, and many of these compounds are present as different stereoisomers [25,26,27]. The assessment of the enantiomeric compositions of chiral terpene mixtures in plant-derived extracts is important in understanding the physiological and ecological roles of the biologically active enantiomers [28,29]. The chiral ratios of specific terpenic molecules may allow for the detection of EO adulteration, which is typically accomplished by the admixture of synthetic aromatic compounds. In this respect, single-dimensional (1D) enantioselective gas chromatography (*e*GC) has been used as one of the most powerful tools for the determination of the enantiomeric composition of chiral terpenes in plant EOs [30,31,32]. In particular, the development of various versatile chiral stationary phases in *e*GC has greatly facilitated the stereo-differentiation of chiral terpenes from a variety of EOs [33,34,35,36]. *e*GC has been successfully used in the past for the authentication of several *Citrus* spp. EOs, including *C. aurantiifolia* [37], *C. reticulata* [37], and *C. limon* [38]. Specifically, the enantiomeric distribution of chiral compounds can be described as enantiomeric excess (EE), enantiomeric ratio (ER), or enantiomeric fraction (EF), with EF as the preferred description of the relative amounts of the enantiomeric pairs [39].

Albeit *e*GC regularly provides sufficient resolution for chiral discrimination of chiral volatile organic compounds in EOs, a few studies have highlighted the applicability of enantioselective comprehensive two-dimensional gas chromatography (*e*GC×GC) for the interference-free ascertainment of ER or EE of volatile racemates in complex sample matrices [40,41]. In a typical *e*GC×GC arrangement, the first-dimension (^1^D) column generally consists of a chiral stationary phase, where chiral molecules are being differentiated into their respective enantiomers, and second-dimension (^2^D) achiral column phases will provide additional separation to address non-specific co-elution that may arise for the target enantiomers. The main advantages of GC×GC over conventional 1D GC correspond to greater peak capacity (i.e., improved resolution), in addition to the structured two-dimensional chromatogram that greatly facilitates peak identification. This high-resolution approach has been recently used for the quality control and/or authentication of *C. aurantium* [42], *C. myrtifolia* [43], and *C. limon* [44] EOs by accurately determining the EF, EE, or ER of chiral terpenic molecules.

Despite the numerous reports on the chiral analysis of *Citrus* spp. EOs, there are only a few studies that examined the enantiomeric composition of chiral terpenes of *C. hystrix* (Kaffir lime), *C. pyriformis* (Ponderosa lemon), and *C. microcarpa* (Calamansi lime) cultivated in Malaysia. Thus, this study aims to evaluate the applicability of cryogen-free thermal modulation-based enantioselective comprehensive two-dimensional gas chromatography–flame ionization detection (*e*GC×GC–FID) method for the stereoisomeric analysis of enantiomers of selected chiral terpenes in *C. hystrix*, *C. pyriformis*, *C. limon*, and *C. microcarpa* leaf EOs. To our knowledge, this is the first application that uses a solid-state modulator to effect the modulation of the ^1^D effluents for the enantiomeric analysis of chiral terpenes in *Citrus* spp. leaf oils. The chromatographic elution and/or separation behavior of different ^1^D chiral phases were investigated to achieve adequate enantio-resolution for the targeted enantiomers, while interfering compounds were further separated in the ^2^D achiral polar phase. EFs of α-pinene, limonene, citronellal, linalool, and terpinen-4-ol in *Citrus* spp. leaf oils were comparatively investigated using 1D *e*GC and *e*GC×GC methods. The prospect of using these chiral ratios to differentiate the analysed *Citrus* spp. leaf oils is discussed.

## 2. Results and Discussion

### 2.1. Enantioselective GC–FID Analysis of Citrus spp. Leaf EOs

**Figure 1:**
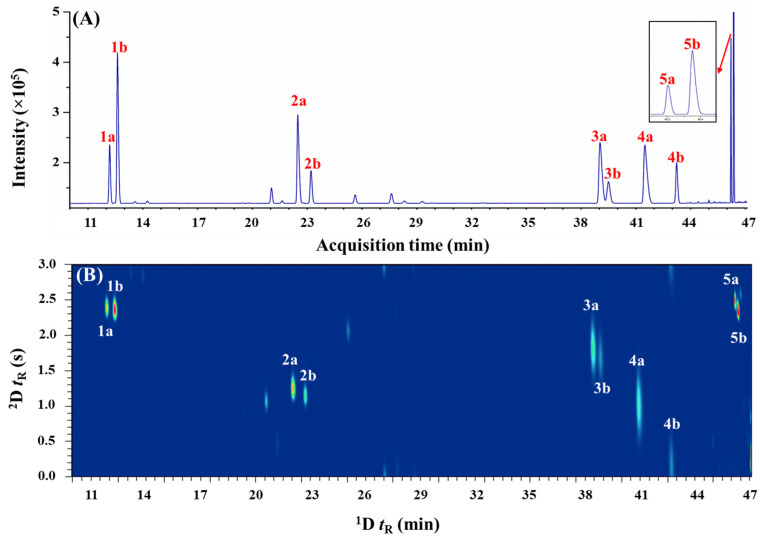
The enantioseparation of standard mixtures using (**A**) *e*GC–FID and (**B**) *e*GC×GC–FID. 1a, (−)-α-pinene; 1b, (+)-α-pinene; 2a, (−)-limonene; 2b, (+)-limonene; 3a, (−)-citronellal; 3b, (+)-citronellal; 4a, (−)-linalool; 4b, (+)-linalool; 5a, (+)-terpinen-4-ol; and (5b), (−)-terpinen-4-ol.

The phytochemical compositions of steam-distilled *C. hystrix*, *C. limon*, *C. pyriformis*, and *C. microcarpa* leaf EOs have been recently reported [45]. α-Pinene, limonene, citronellal, linalool, and terpinen-4-ol were selected for the current study as these chiral monoterpenes (except citronellal) were found to be potentially present in all the studied *Citrus* spp. leaf oils. To justify the requirement of higher resolution *e*GC×GC separation approach for the enantiomeric analysis of *Citrus* spp. leaf oils, preliminary chiral analysis was first conducted using a one-dimensional *e*GC approach. It is known that the separation of enantiomorphic pairs of chiral terpenoids can be achieved using a chiral stationary phase, which typically consists of cyclodextrin derivatives solubilized in polysiloxane. Theoretically, enantiomorphic complexes formed by chiral monoterpenes and cyclodextrin derivatives result in different activity coefficients (i.e., different enantioseparation factors) that result in the separation of the enantiomers. As the chiral recognition is dependent on the chiral selectors within the stationary phase, different substituted β-cyclodextrin (β-CD) phases were evaluated. Amongst the five chiral monoterpenes studied, diacetyl tertbutylsilyl β-cyclodextrin (DAC-β-CD) phase resolves one pair of enantiomers, two pairs are resolved in diethyl tertbutylsilyl β-cyclodextrin (DET-β-CD), while dimethyl tertbutylsilyl β-cyclodextrin (DMT-β-CD) phase resolves only four enantiomeric pairs. All of the chiral terpenes were enantioseparated into their respective enantiomers using dimethyl pentyl-β-cyclodextrin (DMP-β-CD), as shown in Figure 1A.

**Figure 2:**
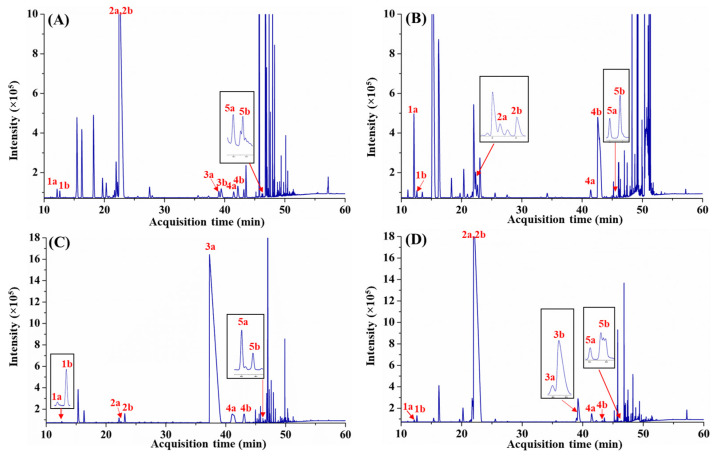
Enantioanalysis of selected chiral monoterpenes in four *Citrus* spp. leaf EOs using eGC–FID. (**A**), *C. limon*; (**B**), *C. microcarpa*; (**C**), *C. hystrix*; and (**D**), *C. pyriformis*. 1a, (−)-α-pinene; 1b, (+)-α-pinene; 2a, (−)-limonene; 2b, (+)-limonene; 3a, (−)-citronellal; 3b, (+)-citronellal; 4a, (−)-linalool; 4b, (+)-linalool; 5a, (+)-terpinen-4-ol; and (5b), (−)-terpinen-4-ol.

The *e*GC–FID method was then applied for the enantiomeric analysis of different *Citrus* spp. leaf EOs. As expected, considerable co-elutions of the targeted enantiomeric pairs (except α-pinene) with other phytoconstituents were observed (Figure 2), making precise determination of the enantiomeric fractions difficult. In particular, it is challenging to accurately quantitate (±)-terpinen-4-ol fractions, as these compounds were significantly obscured by other compounds with resolution (Rs) < 0.7 (e.g., (−)-terpinen-4-ol for *C. limon* and *C. pyriformis*). Additionally, the enantiomeric assessment of limonene enantiomers for *C. microcarpa* is also compromised by the partial overlap of the (+)-limonene with an unassigned component of the oil (Figure 2B). Clearly, insufficient peak capacity and phase selectivity to separate the targeted optical isomers from other phytoconstituents within the leaf oils will result in ambiguities for the accurate determination of the enantiomeric fraction or enantiomeric excess specific to these optically active antipodes.

### 2.2. eGC×GC–FID Analysis of Citrus spp. Leaf EOs

**Figure 3:**
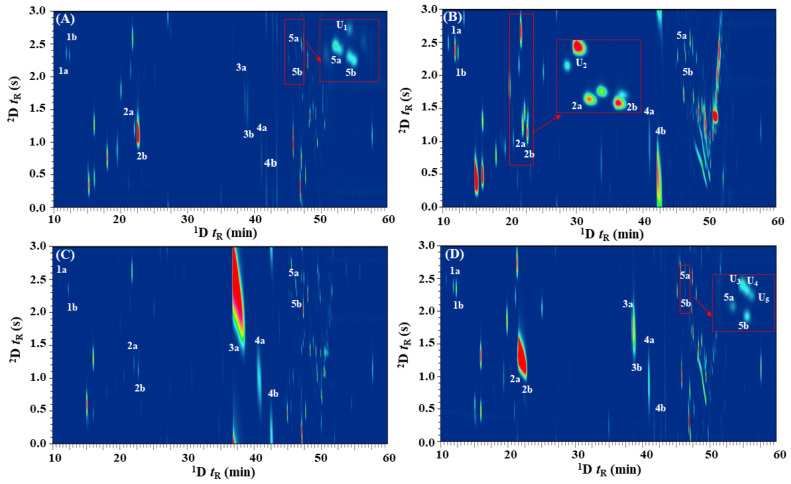
The enantioanalysis of chiral monoterpenes in *Citrus* spp. leaf EOs using *e*GC×GC–FID. (**A**) *C. limon*; (**B**) *C. microcarpa*; (**C**) *C. hystrix*; and (**D**) *C. pyriformis*. 1a, (−)-α-pinene; 1b, (+)-α-pinene; 2a, (−)-limonene; 2b, (+)-limonene; 3a, (−)-citronellal; 3b, (+)-citronellal; 4a, (−)-linalool; 4b, (+)-linalool; 5a, (+)-terpinen-4-ol; and (5b), (−)-terpinen-4-ol.

An enantioselective GC×GC–FID approach that provides better separation performance was evaluated for the enantioresolution of the chiral terpenes in *Citrus* spp. leaf oils. A combination of chiral × polar column sets were used to achieve the appropriate separation (Figure 1B) that approximate the difference in solutes vapor pressure and chiral recognition-based interactions, followed by polarity basis in ^2^D. Albeit theoretically, the ^1^D and ^2^D have different separation mechanisms, but it is important to note that partial correlation between the DMP-β-CD and SUPELCOWAX-10 phases will still exist, as the vapor pressure of volatile constituents still plays a notable role for all GC separations. The alternative arrangement of performing enantioseparation in ^2^D (i.e., GC×*e*GC; polar × chiral) was not investigated due to the difficulties in achieving successful stereoanalysis on the short ^2^D enantioselective column. For *e*GC×GC analysis, it is important to note that peak volume (i.e., the sum of responses across all modulated traces) is the only reliable measure to obtain the enantiomeric distributions. The ^1^D enantioselective column provided resolution of the enantiomers, and during the modulation events, different relative proportions of each enantiomer were sampled and rapidly delivered as pulsed peaks to the ^2^D column for additional achiral separation to resolve any potential interfering components. In this instance, it is important to note that for enantiomorphic pairs that displayed low enantioresolution in the ^1^D (e.g., citronellal; Rs < 1.5), the number of modulations across the ^1^D peak will be important for precise quantification of integrated responses of enantiomers. Thus, the effects of modulation ratio (M_R_) on EF were briefly investigated using citronellal enantiomers (^1^*w*_h_ ~ 11.4 s; ^1^*w*_b_ ~ 19.2 s) by varying the modulation periods (P_M_). The results showed that a P_M_ of 3s provide an M_R_ (calculated using M_R_ = (*w*_h_ × 1.6985)/P_M_) [46] value of ~6.4 that is adequate for accurate determination of EFs by reducing the relative proportion of “shared modulated peak” that constituted indeterminate composition of (+)- and (−)-citronellal. The “shared modulated peak” can be explained by the “intersect” region (i.e., incomplete separation in ^1^D) of both (+) and (−) enantiomers being sampled within the same modulation event and re-injected into the ^2^D SUPELCOWAX-10 column. At this point, the non-enantioselective ^2^D column provides no resolution of the (+) and (−) antipodes. Overall, a DMP-β-CD × SUPELCOWAX-10 column combination with a P_M_ of 3s provides satisfactory separation of all the chiral monoterpenes from other potential interfering phytoconstituents within *Citrus* spp. leaf oils (Figure 3). In comparison to the *e*GC approach (Figure 2), the gain in phytoconstituent coverage in the 2D separation space can be readily observed. Albeit not using cryogens (liquid CO_2_ or N_2_) to modulate the ^1^D effluents, the peak focusing and compression effects of the solid-state modulator (SSM) were noteworthy, as evidenced by the narrow *w*_b_ of the modulated peaks (0.11–0.57 s) as compared to the *w*_b_ for *e*GC (3.12–24.42 s). Despite the SSM having an achiral modulation column to interface the ^1^D enantioselective and ^2^D SUPELCOWAX-10 columns, no significant loss of ^1^D enantioresolution or band broadening (average modulated *w*_b_ of 0.29 s) issue was observed. Citronellal and linalool enantiomers displayed relatively broad modulated peaks (*w*_b_ ~ 0.50 s) as compared to others (average *w*_b_ ~ 0.15 s) due to their strong retention in the ^2^D polar stationary phase. From the obtained contour plots (Figure 3), it can be readily observed that interfering compounds that affect the accurate quantitation of peak volumes for respective chiral monoterpenes have been resolved via ^2^D separation. For instance, the (−)-terpinen-4-ol (Figure 3A) in *C. limon* leaf oil was successfully separated from an unknown component U1, with ^2^t_R_ of 2.28 s and 2.65 s respectively. The (−)-limonene (^2^t_R_ of 1.25 s) in *C. microcarpa* was resolved from an unknown compound U2 (Figure 3B; ^2^t_R_ of 2.65 s), while three unidentified compounds U3, U4, and U5, that co-eluted with (−)-terpinen-4-ol (^2^t_R_ of 2.33 s; approximate a quadruple component broad peak in *e*GC) in *C. pyriformis* were satisfactorily eluted at different retentions in ^2^D with ^2^t_R_ of 2.72 s, 2.65 s, and 2.59 s, respectively. These results demonstrated that *e*GC×GC is a promising alternative to the one-dimensional *e*GC method that provides high-resolution enantioanalysis of chiral terpenes in *Citrus* spp. leaf oils.

### 2.3. Enantiomeric Distribution of Selected Chiral Monoterpenes in Citrus spp. Leaf EOs

The enantiomeric compositions obtained for α-pinene, limonene, citronellal, linalool, and terpinen-4-ol are crucial in safeguarding the quality and authenticity of *Citrus* spp. leaf oils, despite the fact that information concerning these aromatic oils remains scarce. Overall, the EFs determined using *e*GC–FID and *e*GC×GC–FID (Table 1) for chiral monoterpenes that do not suffer co-elutions (e.g., α-pinene) were generally comparable across all *Citrus* spp. oil samples with a variation of <0.7%. Thus, it is readily recognised that a simple 1D *e*GC–FID method should be adequate for enantiomeric analysis of less complex samples that comprise a lesser degree of co-elutions. However, compounds with significant overlapping with other matrix components (e.g., (−)-terpinen-4-ol in *C. limon*) exhibited significant differences in EF (variation of ~11.8%), suggesting possible overestimation or underestimation of enantiomeric compositions using the *e*GC–FID approach. Thus, the high complexity of *Citrus* spp. leaf EOs (Figure 2) with extensive chemical diversity of secondary compounds justified the need for a higher resolving power *e*GC×GC–FID method for correct estimation of enantiomeric excess. Results indicated that (+)-limonene consistently predominated in all analyzed *Citrus* spp. oils with EF > 60%, in which *C. limon* and *C. pyriformis* oils exhibited approximately similar EEs of 96.8% and 97.8%, respectively. In the case of citronellal, the (−) antipode was found to be enantiomerically pure (i.e., EE of 100%) in *C. hystrix* oil, while *C. limon* and *C. pyriformis* have the (+) antipode as the major enantiomer (EF > 65%). This compound was not found in *C. microcarpa* oil. Interestingly, α-pinene, linalool, and terpinen-4-ol displayed different EF across all analyzed *Citrus* species. (+)-α-Pinene predominated in *C*. *hystrix* and *C. pyriformis* (EF of 91.1% and 76.5%, respectively), while (−)-α-pinene was the major enantiomer in *C. limon* (55.5%) and *C. microcarpa* (92.1%). For linalool, the (−)-antipode predominates in *C. limon* (52.5%), *C*. *hystrix* (67.2%), and *C. pyriformis* (91.9%), while (+)-analogue dominate in *C. microcarpa* (95.9%). *C. limon* and *C*. *hystrix* displayed enantiomeric compositions of 53.3% and 70.4% for (+)-terpinen-4-ol, which are different from *C. microcarpa* (33.6%) and *C. pyriformis* (38.9%). In summary, notable differences were observed for the enantiomeric distributions of the investigated chiral compounds of *C. limon*, *C. microcarpa*, *C. hystrix*, and *C. pyriformis* leaf EOs. This suggested the potential for developing a stereoisomer distribution database to differentiate the analyzed *Citrus* leaf oils according to their species. Nevertheless, a more thorough study covering a larger representative sample size of *Citrus* spp. leaf oils that further evaluates the influences of geographical origin, harvesting period, and extraction method is warranted to validate the practicability and reliability of using chiral terpene distribution as a reference for differentiation of leaf oil from dissimilar *Citrus* species across different countries.

## 3. Materials and Methods

### 3.1. Chemical and Reagents

(+)-α-Pinene (98%), α-pinene (98%), (*S*)-(−)-limonene (96%), dipentene, (*S*)-(−)-citronellal (96%), (±)-citronellal (≥95%), (−)-linalool (≥95%), linalool (97%), (−)-terpinen-4-ol (≥95%), and terpinen-4-ol (≥95%) were purchased from Sigma-Aldrich (Darmstadt, Germany). HPLC-grade *n*-hexane was supplied by QREC (Asia) Sdn. Bhd. (Selangor, Malaysia).

### 3.2. Citrus Leaf EO Samples

The *Citrus* leaves were sampled from selected plantation areas located at Batu Ferringhi, Penang (*C. pyriformis*), Gelugor, Penang (*C. microcarpa*), and Gemencheh, Negeri Sembilan (*C. hystrix* and *C. limon*). The *Citrus* leaf EOs were extracted by steam distilling the foliage of the *Citrus* plant for 3 h. The collected leaf oils were stored refrigerated in a glass vial at 4 °C until further analysis. Prior to *e*GC analysis, the leaf oils were diluted in *n*-hexane to the desired concentrations (0.5%, 1.0%, and 2.0% *v*/*v*).

### 3.3. eGC–FID System

*e*GC analyses were conducted on an Agilent Technologies 7890B GC system (Agilent Technologies, Santa Clara, CA, USA) equipped with a flame ionization detector (FID), a 7693A autosampler, and a split/splitless inlet. The enantioseparation was evaluated using a series of enantioselective columns (DMT-β-CD, DET-β-CD, DMP-β-CD, and DAC-β-CD) supplied by MEGA S.r.l. (Legnano, Italy), and a MEGA-DEX DMP-β-CD capillary column of dimensions 25 m × 0.25 mm I.D. × 0.25 μm film thickness (d_f_) was selected for further study. A range of oven ramp rates was investigated to determine the ramp rate that provides optimum enantioseparation and a shorter analysis time. The chromatographic conditions used were: oven temperature program of 40 °C (hold 2 min) to 60 °C at 25 °C min^−1^, followed by 1 °C min^−1^ to 80 °C, 0.5 °C min^−1^ to 90 °C, 20 °C min^−1^ to 130 °C, and 10 °C min^−1^ to 170 °C; injector temperature of 210 °C; FID temperature of 210 °C; helium (99.999%) as the carrier gas at a constant flow rate of 1.0 mL min^−1^ (approximately 26 cm s^−1^); injection volume of 1 µL; and a split ratio of 20:1.

### 3.4. eGC×GC–FID System

Separations were conducted on an Agilent 7890B GC system equipped with a FID, a 7693A autosampler, and a split/split-less inlet. The system was retrofitted with a solid-state thermal modulation system (SSM 1810, J&X Technologies, Shanghai, China). The chromatographic separation was performed using a MEGA-DEX DMP-β-CD (MEGA, Legnano, Italy; 25 m × 0.25 mm I.D. × 0.25 μm d_f_) as the ^1^D column, and a SUPELCOWAX-10 (Supelco, Bellefonte, PA, USA) of dimensions 1.0 m × 0.1 mm I.D. × 0.1 μm d_f_ was used as the ^2^D column, connected by a SV series modulation column (J&X Technologies, Shanghai, China) coated with proprietary phase (no further elaboration by the manufacturer). A deactivated press-tight connector (Restek Corp., Bellefonte, PA, USA) was used to connect the capillary columns (^1^D and ^2^D) and the modulation column. The chromatographic conditions used were: oven temperature program of 40 °C (hold 2 min) to 60 °C at 25 °C min^−1^, followed by 1 °C min^−1^ to 80 °C, 0.5 °C min^−1^ to 90 °C, 20 °C min^−1^ to 130 °C, 10 °C min^−1^ to 170 °C, and 25 °C min^−1^ to 210 °C (hold 15 min); injection volume of 1 µL; injector temperature of 210 °C; detector temperature of 210 °C; sampling frequency of 100 Hz; helium (99.999%) at a flow rate of 1.0 mL min^−1^; injection volume of 1 μL; and a split ratio of 20:1. The entry and exit hot zones (i.e., micathermic heaters) of the modulator permit temperature programming from 50 °C to 320 °C. The cold trapping zone began at 9 °C, which was then ramped down to −51 °C at a rate of −50 °C/min to facilitate the trapping and focusing of the ^1^D effluents. The modulation was performed using a P_M_ of 3 s, although other sampling durations were also evaluated.

### 3.5. Data Handling

Data processing was performed using Agilent Mass Hunter Qualitative Analysis 10.0 (Agilent Technologies, Santa Clara, CA, USA) for *e*GC–FID and *e*GC×GC–FID. The modulation platform was controlled using SSCenter software (v.2.6, J&X Technologies, Shanghai, China). Compound identification was performed based on the co-injection of respective standards to confirm their retentions in the *e*GC and *e*GC×GC methods. Acquired data from Agilent Mass Hunter Qualitative Analysis 10.0 software was exported and further processed using Origin 8 (OriginLab Corporation, Northampton, MA, USA). Canvas software (v.1.8, J&X Technologies, Shanghai, China) was used to generate the contour plots and facilitate further data processing.

## 4. Conclusions

This study evaluates the applicability of *e*GC–FID and *e*GC×GC–FID approaches for assessing the enantiomeric compositions of α-pinene, limonene, citronellal, linalool, and terpinen-4-ol in steam-distilled leaf oils derived from *C. hystrix*, *C. limon*, *C. pyriformis*, and *C. microcarpa*. Enantioseparation of all the targeted chiral terpenes from other interfering volatile secondary compounds was achievable using *e*GC×GC–FID with DMP-β-CD as the ^1^D and SUPELCOWAX-10 as the ^2^D. A modulation period of 3 s was found to provide sufficient modulations (as defined by M_R_) across the ^1^D effluents, allowing better accuracy for the determination of EFs. The summation of all the modulated peak volumes for each enantiomeric pair provided EF values close to those obtained from eGC–FID, provided that there are no significant co-elutions (e.g., α-pinene) with other phytoconstituents. On comparing the results obtained by *e*GC–FID with *e*GC×GC–FID, notable differences (≥11.8%) were observed for the EFs of terpinen-4-ol in *C. limon* and *C. pyriformis* leaf oils that showed considerable overlap with interfering components in *e*GC analysis. (+)-Limonene was identified as the predominant enantiomer (60.3–98.9%) in all *Citrus* spp. leaf oils, while (−)-linalool was the major enantiomer in *C. limon* (52.5%), *C. hystrix* (67.2%), and *C. pyriformis* (91.9%). For terpenin-4-ol, (+)-antipode was the predominant isomer in *C. limon* (53.3%) and *C. hystrix* (70.4%). (+)-α-Pinene was the major antipode in *C. hystrix* (91.1%) and *C. pyriformis* (76.5%). Notable differences were observed for (−)-citronellal in which *C. hystrix* was found to contain pure (−) isomer (EE of 100%), while both *C. limon* (65.4%) and *C. pyriformis* (91.0%) have the (+) antipode as the predominant enantiomer, and *C. microcarpa* indicates the absence of citronellal. The achieved results indicated differences in terms of the enantiomeric distributions of these chiral terpenes in different *Citrus* spp. leaf oils, which might be useful as references for *Citrus* EO producers, merchants, and consumers for quality control and further potential authentication purposes.

## Figures and Tables

**Table 1:** The enantiomeric composition (%) of selected chiral monoterpenes analyzed in *Citrus* spp. leaf EOs using the proposed *e*GC–FID and *e*GC×GC–FID methods.

Chiral Monoterpenes	Antipode	Enantiomeric Composition (%) of *Citrus* spp. Leaf EO			
		*C. limon*	*C. hystrix*	*C. microcarpa*	*C. pyriformis*
α-pinene	(−)	55.5 ^1^ (55.9) ^2^	8.9 (9.4)	92.1 (92.2)	23.5 (24.2)
	(+)	44.5 (44.1)	91.1 (90.6)	7.9 (7.8)	76.5 (75.8)
limonene	(−)	1.6 (1.3)	39.7 (31.5)	38.9 (35.1)	1.1 (0.4)
	(+)	98.4 (98.7)	60.3 (68.5)	61.1 (64.9)	98.9 (99.6)
citronellal	(−)	34.6 (36.0)	100.0 (100.0)	n.d. ^3^ (n.d.)	9.0 (8.7)
	(+)	65.4 (64.0)	0.0 (0.0)	n.d. ^3^ (n.d.)	91.0 (91.3)
linalool	(−)	52.5 (53.0)	67.2 (67.1)	4.1 (3.8)	91.9 (92.1)
	(+)	47.5 (47.0)	32.8 (32.9)	95.9 (96.2)	8.1 (7.9)
terpinen-4-ol	(+)	53.3 (41.5)	70.4 (73.1)	33.6 (30.4)	38.9 (23.6)
	(−)	46.7 (58.5)	29.6 (26.9)	66.4 (69.6)	61.1 (n.a. ^4^)

^1^—Stereoisomeric analysis performed using *e*GC×GC–FID. ^2^—Stereoisomeric analysis performed using *e*GC–FID. ^3^—n.d., compound not detected. ^4^—n.a., EF value non-assessable due to overlapping of the enantiomer with interfering compounds.

## Data Availability

Data are available upon request.

## Sample Availability

*Citrus* spp. EOs are available upon request.
